# Statins and endocrine resistance in breast cancer

**DOI:** 10.20517/cdr.2020.112

**Published:** 2021-06-19

**Authors:** Tara Hyder, Juan Luis Gomez Marti, Azadeh Nasrazadani, Adam M. Brufsky

**Affiliations:** ^1^University of Pittsburgh Physicians, Pittsburgh, PA 15213, USA.; ^2^Department of Pathology, University of Pittsburgh School of Medicine, Pittsburgh, PA 15213, USA.; ^3^UPMC Hillman Cancer Center, Magee Women’s Hospital, Pittsburgh, PA 15213, USA.

**Keywords:** Statin, HMGCR, endocrine resistance, autophagy, PI3K, mTOR

## Abstract

Most breast cancers are hormone-receptor positive (HR^+^). However, more women eventually die from HR^+^ breast cancer than from either HER2^+^ or triple negative breast cancer. Endocrine therapies continue to be the mainstay of treatment. In 40% of these cases, recurrences in early-stage disease and progression in the metastatic setting are largely a function of the development of endocrine resistance. A multitude of mediators and pathways have been associated with endocrine resistance in breast cancer including the mevalonate pathway, which is integral to cholesterol biosynthesis. The mevalonate pathway and the downstream activation of associated cytoplasmic pathways including PI3K-AKT-mTOR and RAS-MEK-ERK have been known to affect cancer cell proliferation, cell survival, cell invasion, and metastasis. These are important mechanisms leading to the inevitable development of endocrine resistance in HR^+^ breast cancer. Statins are a class of drugs that inhibits HMG-CoA reductase, an enzyme in the mevalonate pathway that plays a central role in cholesterol production. *In vitro* and *in vitro* studies suggest that the role of statins in blocking the mevalonate pathway effectively disrupts downstream pathways involved in estrogen receptor expression and cellular processes such as cell survival, proliferation, stress, cell cycle, inhibition of apoptosis, and autophagy. Overcoming these key mechanisms heralds a role for statins in the prevention of endocrine resistance.

## Endocrine resistance in breast cancer

Breast cancer is the most frequently diagnosed cancer and the leading cause of cancer-related death in women throughout the world^[1]^. Roughly two-thirds of breast cancer patients have a hormone-receptor positive (HR^+^) disease for which endocrine therapy is the mainstay of treatment. Endocrine therapy agents function by suppressing the expression of estrogen, in many cases by antagonizing the estrogen receptor (ER). Selective ER modulators (SERMs) such as tamoxifen competitively bind ER, forming an inactive complex that blocks estrogen effect on breast tissue^[2]^. These drugs are demonstrated to be effective in both premenopausal and postmenopausal women, although are preferentially utilized in premenopausal women. Aromatase inhibitors (AIs) such as letrozole, anastrozole, and exemestane aim to reduce peripheral estrogen production through inhibition of aromatase, which facilitates its conversion. AIs are primarily used in postmenopausal women, although there is a role for them in conjunction with GnRH analogues in the premenopausal setting.

A major challenge in treating HR^+^ breast cancer lies in overcoming endocrine resistance, which occurs in approximately 40% of patients^[3]^. Primary endocrine resistance is defined as a relapse within 2 years of adjuvant endocrine treatment or disease progression during the first 6 months of first-line endocrine therapy for advanced or metastatic breast cancer (MBC)^[4]^. Secondary resistance is defined in early breast cancer as a relapse that occurs after at least two years of endocrine therapy and during or within the first year of completing adjuvant endocrine therapy. In advanced or MBC, secondary resistance is defined as disease progression after more than 6 months of endocrine therapy.

Multiple mechanisms of endocrine resistance have been identified including deregulation of the ER signaling pathway, alteration of apoptosis and cell cycle regulation, and the overdrive of pro-proliferative pathways^[5-8]^. One potential pathway implicated in the development of endocrine resistance includes the mevalonate pathway, which is involved in the synthesis of cholesterol and isoprenoids such as geranylgeranyl pyrophosphate (GGPP) and farnesyl pyrophosphate. As a result of this process, 3-hydroxy-3-methyl-glutaryl-CoA reductase (HMGCR) catalyzes the production of mevalonate from 3-hydroxy-3-methyl-glutaryl-CoA. Notably, increased expression of HMGCR has been correlated with increased tumor aggression and poorer prognosis^[9]^. This has led to an increased and newfound interest in statins, a class of drugs known to inhibit HMGCR and classically utilized for the management of hyperlipidemia. Various studies using a range of tumor cell lines have demonstrated anti-angiogenic, anti-proliferative, and pro-apoptotic properties of statins^[10,11]^. More importantly, statin intake has been associated with a decrease in breast cancer recurrence^[12,13]^. Herein, we review literature supporting the role for statins in the prevention of endocrine resistance and breast cancer recurrence.

## Cholesterol biosynthesis and metabolism drives tamoxifen resistance

**Figure 1:**
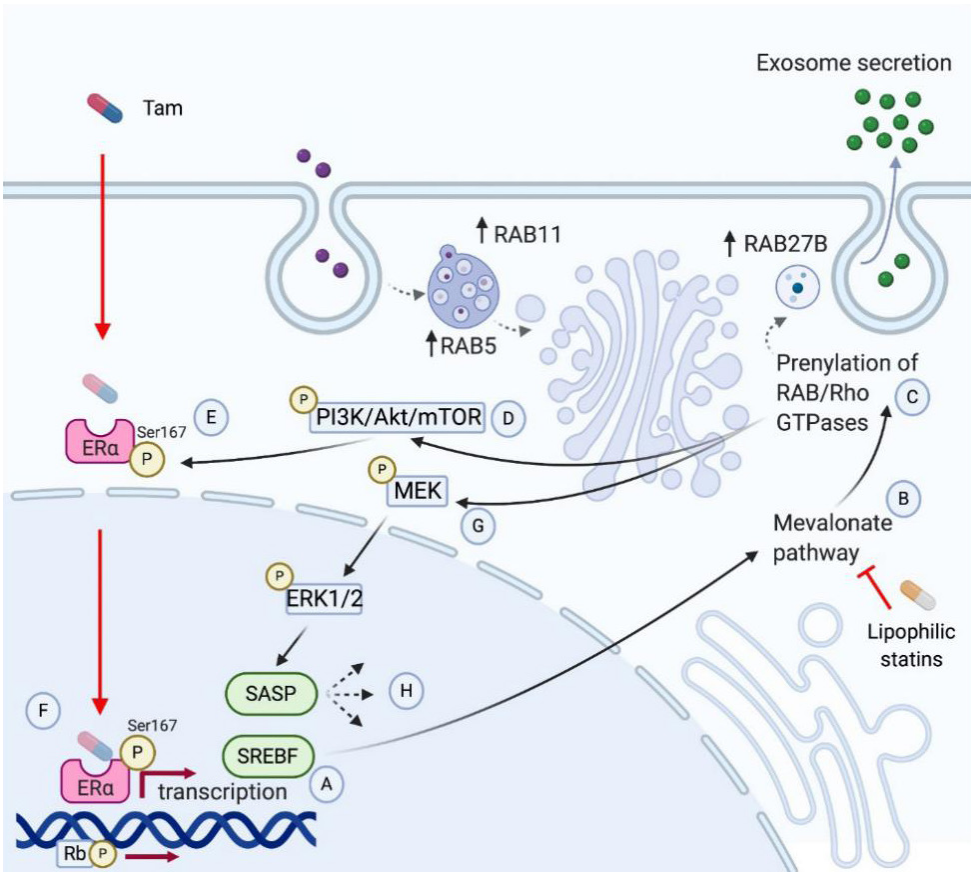
Mechanisms of endocrine resistance in breast cancer. Tamoxifen resistance has been associated with increased expression of the transcription factor SREBF (A). SREBF activates the transcription of mevalonate pathway (MVP) genes, like *HMGCR* (B). Activation of the mevalonate pathway induces prenylation of small GTPases such as Rho, Ras, or Rab (C). Ras prenylation leads to phosphorylation of PI3K (D), which activates Akt and mTOR to phosphorylate the residue, Ser167, of the estrogen receptor (ERa), which decreases sensitivity to tamoxifen (E). This leads to continued Era-derived transcription and phosphorylation of Rb, accelerating G1/S cell cycle transition (F). Ras phosphorylation also activates MEK (G), which activates ERK1/2. The latter is associated to a senescence-associated secretory phenotype (SASP) (H). Created with Biorender.com.

Transcriptomic analysis of tamoxifen-resistant cell lines have shown increased expression of genes involved in the cholesterol biosynthesis pathway [Figure 1]. Specifically, genes associated with sterol regulatory element-binding factor (SREBF) activation were found to be upregulated in tamoxifen-resistant T47D cells^[14]^. SREBF is a transcription factor and primary activator of the mevalonate pathway, thereby suggesting that transcriptional reprogramming of resistant cells may be occurring via mevalonate pathway intermediaries. A recent study showed that AI-resistant cells exhibited a higher degree of expression of small Rab GTPase family proteins. This upregulation was also reflected by a remarkable increase in exosome production, meaning that prolonged endocrine therapy may lead to resistance not only by increasing autophagosome formation but also by increased release of small extracellular vesicles. Proteomic analysis demonstrated a 2-fold increase in vesicle-mediated transport. Of these, RAB27B, RAB5, and RAB11 were found to be significantly upregulated^[15]^. The relevance of these small GTPases relies on their roles in tumor invasion and metastasis, which have previously been reported elsewhere^[16-18]^. Rab27B has been found to promote G1 to S phase transition, proliferation, and invasion *in vitro*. *In vitro*, increased *Rab27B* expression promoted tumor invasion and lymph node metastasis in an orthotopic model, primarily via geranylgeranyl diphosphate^[16]^. By in silico analysis, Rab5A is found to be associated with poor prognosis in HR^+^ breast cancer [HR = 1.3 (1.01-1.6), *P* = 0.037] in multivariate analysis among tumors with *Rab5A* expression > 75% percentile^[17]^. *Rab5A* expression is also induced by hypoxia^[19]^. Interestingly, the hypoxic marker, HIF-1a, is upregulated in letrozole-treated tumors^[20]^. Lastly, *Rab11* is involved in controlling cell surface expression of integrin β1, which triggers mechanotransduction signals within the extracellular matrix, allowing for metastatic cell seeding. Lipophilic statins impair Rab11b association and activity in the cell membrane, preventing breast cancer cells from adapting to the brain microenvironment and consequent seeding^[18]^. Lastly, free cholesterol and lipid droplets have been noted to accumulate in lysosomes of T47D tamoxifen-resistant cells, reflecting impaired lipid metabolism due to deficiencies in membrane permeability^[14]^.

Aberrant mechanisms within the phosphoinositide 3-kinases (PI3K) pathway are additionally implicated in tamoxifen resistance. Ras prenylation leads to phosphorylation of PI3K. The PI3K pathway is the most frequently implicated pathway in breast cancer^[21]^. Activation of PI3K results in the production of phosphatidylinositol 3,4,5-triphosphate (PIP_3_) and subsequent recruitment of AKT protein into the cell membrane. The activation of AKT and the subsequent intracellular cascade of phosphorylation of other proteins, including mammalian target of rapamycin (mTOR), is a potent driver of cell cycle progression and survival. Additionally, ER phosphorylation at Ser_167_ by AKT leads to ligand-independent activation of Erα, a potent mechanism in the estrogen-independent growth of breast cancer cells^[22]^. Multiple studies have demonstrated how the overactivation of the PI3K/AKT/mTOR pathway causes resistance to endocrine treatments such as tamoxifen and aromatase inhibitors. In one study, MCF-7 cells were infected with a retrovirus encoding a constitutively active *AKT* gene, ΔAkt-1(CA)^[23]^. The effects of the hormonal drug, 4-hydroxytamoxifen (4HT), were compared between the ER^+^ MCF-7 and MCF7/ΔAkt-1(CA). It was found that the MCF7/ΔAkt-1(CA) cells were approximately 4.3-fold more resistant to the effects of 4HT than MCF-7 cells, implicating the importance of *AKT* expression on the efficacy of tamoxifen in breast cancer.

Activation of the PI3K/AKT/mTOR pathway has also been implicated in the resistance to aromatase inhibitors in breast cancer lines. In one study, the molecular mechanisms involved letrozole-resistant (LR) cell clones from ER^+^ aromatase-expressing breast cancer cell lines, MCF-7/AROM-1^[24]^. In the LR cell line, there was an upregulation of the PI3K/AKT/mTOR pathway, indicated by increased phosphorylation of AKT/mTOR and their downstream substrates, and an increase in Erα protein expression in these cells. In the same study, as a proof of concept *in vitro*, nine patients receiving letrozole alone in a neo-adjuvant setting for six months, developed an early increase of tumor mass in 3 months after an initial response to the drug. This was evidenced by breast ultrasound and *Ki67* expression; the latter was done at baseline, after 14 days as a marker for early response, and at tumor progression. Paired tumor sections from both pre-treatment and post-treatment showed that there was significant upregulation of the *PI3KCA*, *AKT1*, and *mTOR* genes and their corresponding protein levels.

## Anticancer effects of statins

Multiple studies have demonstrated the role of statins in overcoming endocrine resistance [Table 1]. Simvastatin (SVA) is a lipophilic statin derived from lovastatin and has been found to mitigate endocrine resistance by a variety of mechanisms. Tamoxifen resistance, specifically, has been associated with the activation of retinoblastoma protein (Rb), an integral regulator of G1/S-phase cell-cycle progression. Deregulation of this pathway is associated with early recurrence of breast cancer following tamoxifen monotherapy^[25]^. Additionally, minichromosome maintenance protein 7 (MCM7) is a part of a larger MCM complex and another key regulator of the cell cycle^[26]^. Knockdown of MCM7 leads to abnormal replication of DNA during the S phase, which in turn activates the DNA damage response in order to halt cell-cycle progression^[27]^. SVA has been shown to downregulate both Rb and MCM7 in two tamoxifen-resistant breast cancer cell lines, MCF7 and T47D, thereby leading to DNA damage. The addition of simvastatin to tamoxifen, however, demonstrates retarded growth of the tamoxifen-resistant cells leading to an induction of apoptosis^[28]^. Given that the increased prenylation signaling downstream of the cholesterol synthesis pathway that arguably contributes to endocrine resistance, the aforementioned data suggest that effective blockade of the mevalonate pathway may aid in re-sensitizing breast cancer cells.

**Table 1:** Mechanisms of statins in prevention of endocrine resistance and cancer recurrence

Study	Statin studied	Lipophilic *vs.* hydrophilic	Mechanism of action
Liang *et al*.^[28]^	Simvastatin + Tamoxifen	Lipophilic	Downregulates expression of *MCM7* leading to DNA damage in tamoxifen-resistant cells
Liu *et al*.^[31]^	Simvastatin	Lipophilic	Reduces isoprenoid intermediates of the mevalonate pathway Reduces activity of Rho GTPases, Rac1 and Cdc42 Inhibits ERK1/2 pathway, which leads to activation of SASP
Moriai *et al*.^[35]^	Lovastatin + Tamoxifen	Lipophilic	Downregulates survivin protein expression, which increases sensitivity to tamoxifen-induced apoptosis
Kusama *et al*.^[38]^	Fluvastatin	Lipophilic	Inhibition of Rho A and Rho C membrane localization, thereby impairing cancer cell migration and invasion into the endothelial cell layer
Liu *et al*.^[39]^	Fatostatin + Tamoxfen	-	Inhibitors of SREBP pathway resulting in decreased tumor cell invasion Degradation of ER protein PI3K-AKT-mTOR signaling disruption leading to apoptosis and autophagy Enhance tamoxifen-induced apoptosis and autophagy Enhanced tamoxifen-induced cell cycle arrest
Iizuka-Ohashi *et al*.^[40]^	Fluvastatin	Lipophilic	Suppression of AKT activation, which decreases apoptotic resistance to MEK inhibitors
Miettinen *et al*.^[44]^	Atorvastatin	Lipophilic	Induces accumulation of autophagosomes and decreased autophagic flux
Shojaei *et al*.^[45]^	Simvastatin + Temozolomide	Lipophilic	Blocks autophagolysosome formation and increased proapoptotic cell death
Misirkic *et al*.^[46]^	Simvastatin	Lipophilic	Upregulation of autophagolysosome-associated LC3-II indicating induction of autophagy
Toepfer *et al*.^[47]^	Atorvastatin	Lipophilic	Upregulation of autophagolysosome-associated LC3-II indicating induction of autophagy
Qi *et al*.^[48]^	Simvastatin	Lipophilic	Upregulation of autophagolysosome-associated LC3-II indicating induction of autophagy

Simvastatin can suppress breast cancer cell proliferation by affecting cellular senescence. Senescence represents the stress response of the cell to factors such as DNA damage and oncogene activation^[29]^. In response to these triggers, the cells acquire a senescence-associated secretory phenotype (SASP) that secretes different cytokines, growth factors, chemokines, and proteases, which promote inflammation and cancer progression^[30]^. *In vitro*, MCF7 breast cancer cells treated with fulvestrant exhibited lesser treatment effects upon co-culture with senescent *vs.* non-senescent cells^[31]^, implicating senescence as a mechanism that leads to endocrine resistance. However, upon treatment of senescent cells with simvastatin, fulvestrant treatment effects were significantly enhanced, suggesting that simvastatin can mitigate the effects of senescent cells on endocrine resistance in breast cancer cells. A possible explanation for this effect involves an inhibitory effect on ERK1/2 pathway activation in SASP, which has been further linked with poor response to hormone therapy in breast cancer patients^[32]^. Rho GTPases, such as Rac1 and Cdc42, which control cell motility, adhesion, and proliferation are additionally dysregulated in SASP^[33]^. SVA appears to mitigate the effects of these key regulators that lead to SASP-induced endocrine resistance.

In a separate study, survivin was identified as a direct inhibitor of caspase-3 and caspase-7, causing a blunting of tamoxifen-induced apoptosis in MCF-7 and ZR-75-1 breast cancer cells^[34,35]^. This effect was diminished with lovastatin pre-treatment by the downregulation of survivin expression, thus increasing apoptosis.

Statins have also been shown to inhibit migration and invasion of breast cancer cells *in vitro* by preventing geranylgeranylation of Rho. The Rho family of proteins consists of GTPases including *Rho A* and *Rho C* that are overexpressed in breast cancer, and implicated in cancer cell migration and invasion^[36]^. To be fully functional, they require post-translational isoprenylation by specific transferases, farnesyltransferase, and geranylgeranyltransferase type I^[37]^. MDA-MB-231 cells previously treated with fluvastatin demonstrate decreased levels of Rho A and Rho C, which in turn leads to inhibition of transendothelial migration and invasion^[38]^. Cell invasion was further found to be impaired in MCF-7, T47D, MDA-MB-231, and MDA-MB-468 cells treated with both fatostatin and tamoxifen, owing to a synergistic effect between the agents and the ability of fatostatin to degrade ER via K-48 linked polyubiquitination. An added benefit of fatostatin in this study contributed to autophagy and apoptosis^[39]^.

Statins may have a role in overcoming resistance to MEK inhibitors such as tramatenib and CH5126766. In a preclinical study, several cancer cell lines including the human breast cancer cells, MDA-MB-231, were treated with a MEK inhibitor, CH5126766, with or without statins^[40]^. There was a dose-dependent reduction in cell growth in cancer cells treated with combined CH5126766 and fluvastatin versus CH5126766 alone. Flow cytometric analysis of the cell cycle demonstrated that treatment with statins induced G1 arrest in MDA-MB-231 cells irrespective of CH5126766 administration. Activation of PI3K-AKT signaling and the subsequent increased expression of SREBPs following inhibition of the MEK pathway plays a role in the apoptotic resistance of cancer cells to MEK inhibitors. The addition of fluvastatin or simvastatin was able to suppress CH5126766-induced activation of AKT. Therefore, the utilization of statins in the blockade of the mevalonate pathway leads to the suppression of AKT activation and a decreased apoptotic resistance to MEK inhibitors.

## Autophagy and statins

Autophagy is a recycling pro-survival process where cells breakdown the utilized cytoplasmic products, which are then incorporated as auto-phagolysosomes and converted into inputs of cell metabolism^[41]^. Induction of autophagy is associated with the development of therapeutic resistance in breast cancer^[42]^. In fact, previous research shows that tamoxifen induces protective autophagy and the eventual induction of endocrine resistance in breast cancer cells, which can become re-sensitized by inhibiting autophagy^[42-43]^.

Autophagy is regulated by the mevalonate pathway in the form of geranylgeranylation of small Rab GTPases. Particularly, Rab11 has been proposed as the main link between the mevalonate pathway and autophagy. Inhibition of the mevalonate pathway with atorvastatin induces accumulation of autophagosomes and reduced autophagic flux^[44]^. Conversely, other studies have suggested that statin use does not block autophagic flux by blocking the mevalonate pathway, but rather interferes in phagolysosome formation^[45]^. Use of simvastatin in combination with temozolomide showed statin-induced accumulation of autophagosomes and enhanced proapoptotic cell death by temozolomide. It is important to note that the opposite has also been reported, suggesting that high dose statins could in fact increase autophagic flux, as measured by LC3II^[46,47]^. However, an increase in LC3II levels and autophagosomes can be seen upon inhibition of autophagic flux^[47]^. Simvastatin has also been shown to induce cytoplasmic accumulation of autophagic vacuoles, which was reversed by the addition of farnesyl and GGPP in a model of amyotrophic lateral sclerosis^[48]^.

## Statin use and outcomes in clinical trials

In a clinical study examining postmenopausal women with early HR^+^ breast cancer receiving adjuvant AI therapy, breast cancer recurrence over a 5-year follow-up period was significantly less in patients concomitantly exposed to a statin^[49]^. The incidence rate per 1000 person-years was 10.12 (95%CI: 6.92-14.28) in statin-exposed patients and 13.40 (95%CI: 12.36-14.51) in the non-exposed group. Any statin exposure was associated with a reduced rate of 5-year breast cancer recurrence [adjusted HR 0.72 (95%CI: 0.50-1.04)]. In the metastatic setting, an open-labelled phase II clinical study is currently recruiting and will evaluate the benefit of atorvastatin addition to standard endocrine therapy in the second line setting (NCT02958852). While *in vitro* and *in vitro* data support an integral role for the mevalonate pathway in the development of resistance to endocrine therapies, larger scale analyses of clinical data are needed to guide the use of lipid metabolism modulators in clinical practice.

## Conclusion

Our review demonstrates the potential role of statins in overcoming endocrine resistance, an inevitable challenge in the treatment of HR^+^ breast cancer. There are several reasons that make statins a practical choice in the treatment of breast cancer. They are commonly prescribed medications used primarily for the management of hyperlipidemia and cardiovascular disease. According to a report by the Center for Disease Control and Prevention, 23.6% and 38.9% of women over 45 or 75 years, respectively were taking statins^[50]^. In addition, because of their widespread popularity in the prevention of both primary and secondary cardiovascular diseases, generic statin medications now cost < $12/month^[51]^. Statins are also generally well tolerated medications, although it is important to acknowledge their limiting side effects including myopathy that precludes its use in a subpopulation of patients. Also, metabolic syndrome and obesity together are linked with nearly 20% of breast cancers, particularly in the post-menopausal setting^[52]^. Treating obesity and associated comorbidities such as hypercholesterolemia has shown to prevent more than 30% of breast cancers. All these factors make statins a logical choice and should prompt clinical trials to further investigate the role of statins in endocrine-resistant breast cancer.

## Declarations

### Copyright

© The Author(s) 2021.

## References

[1] DeSantis CE, Ma J, Gaudet MM (2019). Breast cancer statistics, 2019.. CA Cancer J Clin.

[2] Patel HK, Bihani T (2018). Selective estrogen receptor modulators (SERMs) and selective estrogen receptor degraders (SERDs) in cancer treatment.. Pharmacol Ther.

[3] Osborne CK, Schiff R (2011). Mechanisms of endocrine resistance in breast cancer.. Annu Rev Med.

[4] Cardoso F, Senkus E, Costa A, Papadopoulos E, Aapro M, André F (2018). 4th ESO-ESMO international consensus guidelines for advanced breast cancer (ABC 4).. Ann Oncol.

[5] Murphy CG, Dickler MN (2016). Endocrine resistance in hormone-responsive breast cancer: mechanisms and therapeutic strategies.. Endocr Relat Cancer.

[6] Mürdter TE, Schroth W, Bacchus-Gerybadze L, German Tamoxifen and AI Clinicians Group (2011). Activity levels of tamoxifen metabolites at the estrogen receptor and the impact of genetic polymorphisms of phase I and II enzymes on their concentration levels in plasma.. Clin Pharmacol Ther.

[7] Gutierrez MC, Detre S, Johnston S (2005). Molecular changes in tamoxifen-resistant breast cancer: relationship between estrogen receptor, HER-2, and p38 mitogen-activated protein kinase.. J Clin Oncol.

[8] Lindström LS, Karlsson E, Wilking UM (2012). Clinically used breast cancer markers such as estrogen receptor, progesterone receptor, and human epidermal growth factor receptor 2 are unstable throughout tumor progression.. J Clin Oncol.

[9] Schointuch MN, Gilliam TP, Stine JE (2014). Simvastatin, an HMG-CoA reductase inhibitor, exhibits anti-metastatic and anti-tumorigenic effects in endometrial cancer.. Gynecol Oncol.

[10] Wong WW, Dimitroulakos J, Minden MD, Penn LZ (2002). HMG-CoA reductase inhibitors and the malignant cell: the statin family of drugs as triggers of tumor-specific apoptosis.. Leukemia.

[11] Graaf MR, Richel DJ, van Noorden CJ, Guchelaar HJ (2004). Effects of statins and farnesyltransferase inhibitors on the development and progression of cancer.. Cancer Treat Rev.

[12] Kwan ML, Habel LA, Flick ED, Quesenberry CP, Caan B (2008). Post-diagnosis statin use and breast cancer recurrence in a prospective cohort study of early stage breast cancer survivors.. Breast Cancer Res Treat..

[13] Ahern TP, Pedersen L, Tarp M (2011). Statin prescriptions and breast cancer recurrence risk: a Danish nationwide prospective cohort study.. J Natl Cancer Inst.

[14] Hultsch S, Kankainen M, Paavolainen L (2018). Association of tamoxifen resistance and lipid reprogramming in breast cancer.. BMC Cancer.

[15] Augimeri G, La Camera G, Gelsomino L (2020). Evidence for enhanced exosome production in aromatase inhibitor-resistant breast cancer cells.. Int J Mol Sci.

[16] Hendrix A, Maynard D, Pauwels P (2010). Effect of the secretory small GTPase Rab27B on breast cancer growth, invasion, and metastasis.. J Natl Cancer Inst.

[17] Frittoli E, Palamidessi A, Marighetti P (2014). A RAB5/RAB4 recycling circuitry induces a proteolytic invasive program and promotes tumor dissemination.. J Cell Biol.

[18] Howe EN, Burnette MD, Justice ME (2020). Rab11b-mediated integrin recycling promotes brain metastatic adaptation and outgrowth.. Nat Commun.

[19] Silva P, Mendoza P, Rivas S (2016). Hypoxia promotes Rab5 activation, leading to tumor cell migration, invasion and metastasis.. Oncotarget.

[20] Jia X, Hong Q, Lei L (2015). Basal and therapy-driven hypoxia-inducible factor-1α confers resistance to endocrine therapy in estrogen receptor-positive breast cancer.. Oncotarget.

[21] Campbell RA, Bhat-Nakshatri P, Patel NM, Constantinidou D, Ali S, Nakshatri H (2001). Phosphatidylinositol 3-kinase/AKT-mediated activation of estrogen receptor alpha: a new model for anti-estrogen resistance.. J Biol Chem.

[22] Miller TW, Hennessy BT, González-Angulo AM (2010). Hyperactivation of phosphatidylinositol-3 kinase promotes escape from hormone dependence in estrogen receptor-positive human breast cancer.. J Clin Invest.

[23] Sokolosky ML, Stadelman KM, Chappell WH (2011). Involvement of Akt-1 and mTOR in sensitivity of breast cancer to targeted therapy.. Oncotarget.

[24] Cavazzoni A, Bonelli MA, Fumarola C (2012). Overcoming acquired resistance to letrozole by targeting the PI3K/AKT/mTOR pathway in breast cancer cell clones.. Cancer Lett.

[25] Bosco EE, Wang Y, Xu H (2007). The retinoblastoma tumor suppressor modifies the therapeutic response of breast cancer.. J Clin Invest.

[26] Maiorano D, Lutzmann M, Méchali M (2006). MCM proteins and DNA replication.. Curr Opin Cell Biol.

[27] Ibarra A, Schwob E, Méndez J (2008). Excess MCM proteins protect human cells from replicative stress by licensing backup origins of replication.. Proc Natl Acad Sci U S A.

[28] Liang Z, Li W, Liu J (2017). Simvastatin suppresses the DNA replication licensing factor MCM7 and inhibits the growth of tamoxifen-resistant breast cancer cells.. Sci Rep.

[29] Campisi J (2013). Aging, cellular senescence, and cancer.. Annu Rev Physiol.

[30] Davalos AR, Kawahara M, Malhotra GK (2013). p53-dependent release of Alarmin HMGB1 is a central mediator of senescent phenotypes.. J Cell Biol.

[31] Liu S, Uppal H, Demaria M, Desprez PY, Campisi J, Kapahi P (2015). Simvastatin suppresses breast cancer cell proliferation induced by senescent cells.. Sci Rep.

[32] Musgrove EA, Sutherland RL (2009). Biological determinants of endocrine resistance in breast cancer.. Nat Rev Cancer.

[33] Debidda M, Williams DA, Zheng Y (2006). Rac1 GTPase regulates cell genomic stability and senescence.. J Biol Chem.

[34] Mandlekar S, Yu R, Tan TH, Kong AN (2000). Activation of caspase-3 and c-Jun NH2-terminal kinase-1 signaling pathways in tamoxifen-induced apoptosis of human breast cancer cells.. Cancer Res.

[35] Moriai R., Tsuji N., Moriai M, Kobayashi D, Watanabe N (2009). Survivin plays as a resistant factor against tamoxifen-induced apoptosis in human breast cancer cells.. Breast Cancer Res Treat.

[36] Hall A (1998). Rho GTPases and the actin cytoskeleton.. Science.

[37] Elson CE, Peffley DM, Hentosh P, Mo H (1999). Isoprenoid-mediated inhibition of mevalonate synthesis: potential application to cancer.. Proc Soc Exp Biol Med.

[38] Kusama T, Mukai M, Tatsuta M, Nakamura H, Inoue M (2006). Inhibition of transendothelial migration and invasion of human breast cancer cells by preventing geranylgeranylation of Rho.. Int J Oncol.

[39] Liu Y, Zhang N, Zhang H (2020). Fatostatin in combination with tamoxifen induces synergistic inhibition in ER-positive breast cancer.. Drug Des Devel Ther.

[40] Iizuka-Ohashi M, Watanabe M, Sukeno M (2018). Blockage of the mevalonate pathway overcomes the apoptotic resistance to MEK inhibitors with suppressing the activation of Akt in cancer cells.. Oncotarget.

[41] Rabinowitz JD, White E (2010). Autophagy and metabolism.. Science.

[42] Samaddar JS, Gaddy VT, Duplantier J (2008). A role for macroautophagy in protection against 4-hydroxytamoxifen-induced cell death and the development of antiestrogen resistance.. Mol Cancer Ther.

[43] Liu J, Yue W, Chen H (2019). The correlation between autophagy and tamoxifen resistance in breast cancer.. Int J Clin Exp Pathol.

[44] Miettinen TP, Björklund M (2015). Mevalonate pathway regulates cell size homeostasis and proteostasis through autophagy.. Cell Rep.

[45] Shojaei S, Koleini N, Samiei E (2020). Simvastatin increases temozolomide-induced cell death by targeting the fusion of autophagosomes and lysosomes.. FEBS J.

[46] Misirkic M, Janjetovic K, Vucicevic L (2012). Inhibition of AMPK-dependent autophagy enhances in vitro antiglioma effect of simvastatin.. Pharmacol Res.

[47] Toepfer N, Childress C, Parikh A, Rukstalis D, Yang W (2011). Atorvastatin induces autophagy in prostate cancer PC3 cells through activation of LC3 transcription.. Cancer Biol Ther.

[48] Qi W, Yan L, Liu Y (2019). Simvastatin aggravates impaired autophagic flux in NSC34-hSOD1G93A cells through inhibition of geranylgeranyl pyrophosphate synthesis.. Neuroscience.

[49] Harborg S, Heide-Jørgensen U, Ahern TP, Ewertz M, Cronin-Fenton D, Borgquist S (2020). Statin use and breast cancer recurrence in postmenopausal women treated with adjuvant aromatase inhibitors: a Danish population-based cohort study.. Breast Cancer Res Treat.

[50] Gu Q, Paulose-Ram R, Burt VL, Kit BK (2014). Prescription cholesterol-lowering medication use in adults aged 40 and over: United States, 2003-2012.. NCHS Data Brief.

[51] Lazar LD, Pletcher MJ, Coxson PG, Bibbins-Domingo K, Goldman L (2011). Cost-effectiveness of statin therapy for primary prevention in a low-cost statin era.. Circulation.

[52] Bhaskaran K, Douglas I, Forbes H, dos-Santos-Silva I, Leon DA, Smeeth L (2014). Body-mass index and risk of 22 specific cancers: a population-based cohort study of 5·24 million UK adults.. Lancet.

